# LRP1 Interacts with the Rift Valley Fever Virus Glycoprotein Gn via a Calcium-Dependent Multivalent Electrostatic Mechanism

**DOI:** 10.3390/biom16010014

**Published:** 2025-12-21

**Authors:** Haonan Yang, Haojin Chen, Wanyan Jiang, Renhong Yan

**Affiliations:** 1 SUSTech Homeostatic Medicine Institute, School of Medicine, Southern University of Science and Technology, Shenzhen 518055, China; 12331447@mail.sustech.edu.cn (H.Y.);; 2 Key University Laboratory of Metabolism and Health of Guangdong, Institute for Biological Electron Microscopy, Southern University of Science and Technology, Shenzhen 518055, China; 3 Institute for Hepatology, National Clinical Research Center for Infectious Disease, Shenzhen Third People’s Hospital, The Second Affiliated Hospital, School of Medicine, Southern University of Science and Technology, Shenzhen 518112, China

**Keywords:** Rift Valley fever virus (RVFV), LRP1, calcium–dependent binding, electrostatic interactions, viral entry

## Abstract

The Rift Valley fever virus (RVFV) is a highly pathogenic, mosquito-borne zoonotic virus that poses a significant risk to livestock, human health, and global public health security. Although RVFV is classified by the World Health Organization (WHO) as a priority pathogen with epidemic potential, no licensed vaccines or effective antiviral therapies are currently available. A limited understanding of the molecular mechanisms of RVFV entry has hindered therapeutic development. Here, we elucidate the molecular basis by which the RVFV envelope glycoprotein Gn recognizes its receptor, low-density lipoprotein receptor-related protein 1 (LRP1). Bio-layer interferometry (BLI) demonstrates that full-length LRP1 directly binds the head domain of Gn with nanomolar affinity in a Ca^2+^-dependent manner. Both LRP1 clusters II (CL II) and IV (CL IV) independently interact with Gn, with CL IV exhibiting stronger affinity, indicating a multivalent recognition mode. Structural modeling using AlphaFold 3 reveals pronounced charge complementarity between basic residues on Gn and acidic, Ca^2+^-coordinated pockets within LRP1. Mutations in key acidic residues in CL IV greatly reduced Gn binding, confirming the essential roles of Ca^2+^ coordination and electrostatic interactions. Collectively, our findings define a Ca^2+^-stabilized, electrostatically driven mechanism for RVFV Gn recognition by LRP1, providing molecular insight into viral entry and a structural framework for the rational design of vaccines and antiviral therapeutics.

## 1. Introduction

The Rift Valley fever virus (RVFV) is a negative-sense RNA virus that is spread by mosquitoes and is part of the *Phenuiviridae* family under the *Phlebovirus* genus [1,2,3]. It is a major zoonotic pathogen that causes severe outbreaks in both livestock and humans [1,4,5]. In ruminants such as cattle and sheep, RVFV infection can cause “abortion storms” in pregnant animals and cause high death rates in young animals, leading to substantial agricultural economic losses [4,6]. In humans, RVFV infection typically presents as a self-limiting febrile illness but can progress to severe manifestations, including hemorrhagic fever, encephalitis, or retinitis, leading to a serious public health concern [1,3]. There are currently no approved vaccines or specific antiviral treatments for either humans or animals, even though there have been repeated RVFV outbreaks in Africa and the Middle East in recent years. The World Health Organization has listed RVFV as a priority pathogen because of its potential as a bioterrorism agent [1,4,7,8,9,10,11]. Furthermore, the molecular mechanisms that trigger RVFV entry into host cells remain poorly known, resulting in challenges to the development of effective antiviral strategies.

The RVFV genome comprises three single-stranded RNA segments: the large (L), medium (M), and small (S). The L segment encodes the viral RNA-dependent RNA polymerase (RdRp), which is responsible for genome replication and transcription. The M segment generates the glycoprotein precursor (GPC) as well as the nonstructural protein NSm. The S segment encodes the nucleocapsid protein (N) and the nonstructural protein NSs. Host signal peptidases cleaved the GPC into two mature envelope glycoproteins, Gn and Gc, which assemble as heterodimers on the viral membrane to form the envelope glycoprotein lattice [12,13,14]. Gn occupies the membrane-distal position, forming the outermost layer of the glycoprotein shell. Gc is in the membrane-proximal position underneath the Gn layer. This arrangement results in the shielding of the Gc fusion loops by Gn under neutral pH conditions, which prevents the hydrophobic fusion loop from premature exposure [14]. Gn is thought to play a key role in triggering the virus’s attachment and binding to host cell receptors [14,15]. Previous studies have indicated that low density lipoprotein receptor related protein 1 (LRP1) acts as a host factor for RVFV [16,17]. LRP1 is a ubiquitously expressed endocytic receptor involved in various physiological processes, including lipid metabolism, apoptotic signaling, and regulation of extracellular protease activity. In addition, LRP1 modulates multiple signaling pathways by internalizing or scaffolding a wide range of ligands and co-receptors, thereby influencing cellular homeostasis and innate immune responses [18,19,20]. The extracellular region of LRP1 consists of four ligand-binding clusters (clusters I–IV). Each cluster has complement-type repeats (CRs) that form Ca^2+^-coordinated acidic pockets, which are crucial for mediating viral entry. Given these functional and structural features, LRP1 has been identified as a key host factor for the RVFV, and understanding its interaction with the virus could provide valuable insights into viral pathogenesis and therapeutic strategy development [21,22,23]. However, the molecular characteristics governing the interaction between RVFV Gn and LRP1 remains poorly known. This includes its Ca^2+^ dependence, the charge distribution of the critical binding interface, the specific domains determining affinity, and the contribution of multivalent binding to overall stability [15,16,17].

In this study, we sought to elucidate the interaction mechanism between LRP1 and the envelope glycoprotein of RVFV. Using bio-layer interferometry (BLI), we quantitatively demonstrate that purified full-length LRP1 directly binds to Gn in a Ca^2+^-dependent manner. Further analysis reveals that both CL II and CL IV of LRP1 could bind to Gn but with affinity lower than that of full-length LRP1. The CL IV exhibits stronger binding than CL II, suggesting that multivalent cooperative effects play a key role in achieving high-affinity binding. Based on AlphaFold 3 structural predictions of the Gn-CL IV complex [24], we reveal the significant charge complementarity between basic regions on the surface of Gn and acidic pockets in LRP1’s repeat modules. Using point mutations at Ca^2+^-coordinating sites and charged residues at the interface, we validated the crucial role of these sites in maintaining binding. These findings point to a model of interaction driven by electrostatic recognition and stabilized by Ca^2+^, with multivalent binding amplifying the affinity. This work provides the molecular-level mapping of the Gn-LRP1 interaction and lays the molecular foundation for the development of novel antiviral strategies aimed at blocking the virus-host interaction.

## 2. Materials and Methods

### 2.1. Protein Expression and Purification

The ectodomain of RVFV glycoprotein Gn (GenBank: AAA47449.1) (154–469 amino acids, aa) and its mutants were cloned into the pCAG vector (Invitrogen, Carlsbad, CA, USA) with an N-terminal signal peptide and a C-terminal FLAG or 6 × HIS tag. The LRP1 (genbank: caa32112.1), including CL II (786–1165 aa) and CL IV (3332–3779 aa), and the truncated and mutated constructs were cloned into the same vector. For the CL IV domain, three truncated constructs were generated, covering the repeats A21–A24 (3332–3491 aa), A25-A28 (3492–3649 aa), and A29–A31 (3652–3778 aa). Single–repeat fusion human FC region protein (CA21–CA31) was also added into the same vector and expressed separately.

Point mutations were introduced into these truncated CL IV constructs, resulting in six mutant constructs: E3356A/D3357A/D3358A, D3374A/D3376A/D3378A, E3517A/D3518A/D3519A, D3556A/D3558A/D3560A, D3595A/D3597A/D3599A, D3633A/D3635A/D3637A. Each mutation was integrated into the corresponding truncated form of CL IV.

The plasmid used for cell transfection was prepared using the GoldHi EndoFree Plasmid Maxi Kit (CWBIO, Taizhou, China). HEK 293 F mammalian cells (Invitrogen) were cultured in SMM 293T-II medium (Sino Biological Inc., Beijing, China) at 37 °C under 5% CO_2_ in a Stackable Constant Temperature Shaker(Jiemei, Suzhou, China). Once the cell density reached 2.0 × 10^6^ cells/mL, the plasmid was transiently transfected into the cells. For the transfection of one liter of cell culture, approximately 1.5 mg of the plasmid was mixed with 3 mg of polyethyleneimines (PEIs) (Yeasen, Shanghai, China) in 50 mL of fresh medium. The mixture was incubated for 15 min before adding it to the cell culture. After 60 h, supernatants were collected by centrifugation at 4000× *g* for 15 min at 4 °C.

The secreted proteins were purified by Ni–NTA affinity resin (Qiagen) or anti-FLAG M2 affinity resin (GenScript, Nanjing, China). Proteins were eluted with buffer containing 300 mM imidazole (For HIS-tagged protein) or 0.2 mg/mL flag peptide (For FLAG-tagged protein), 25 mM HEPES (pH 7.5), and 150 mM NaCl. The eluent was then concentrated and injected to size-exclusion chromatography (Superose 6 Increase 10/300 GL, (Cytiva, Marlborough, MA, USA) in buffer containing 25 mM HEPES (pH 7.5), 150 mM NaCl. The peak fractions were collected and concentrated for analysis.

Native LRP1 was purified from porcine lung by affinity chromatography using related receptor protein (RAP). Endogenous LRP1 was captured by immobilized RAP, washed and eluted with buffer containing 25 mM HEPES (pH 7.5), 150 mM NaCl, 2 mM CaCl_2_ and 0.02%DDM. LRP1 was further purified by SEC with the same buffer. After the initial affinity purification, RAP-LRP1 complex was loaded onto Ni–NTA affinity resin (Qiagen, Hilden, Germany), and RAP was dissociated by acid washing at pH 5.5 to obtain functionally active LRP1. The eluted LRP1 fraction was immediately neutralized and buffer-exchanged to physiological pH prior to subsequent binding assays.

### 2.2. Size-Exclusion Chromatography and Western Blot

In order to evaluate the interaction between LRP1 and Gn (154–469 aa), three groups of experiments were carried out using SEC. Full length LRP1 and Gn (154–469 aa) were incubated at 4 °C for 1 h in binding buffer (25 mM HEPES, pH 7.5, 150 mM NaCl, 2 mM CaCl_2_) with a molar ratio of 1:1.2. The mixture was placed on a Superose 6 increase 10/300 GL column (cytiva, Marlborough, MA, USA) balanced with the same buffer. The fractions corresponding to the potential complex peaks were analyzed by SDS-PAGE and Western blotting using the polyclonal antibody against LRP1 (Proteintech, Wuhan, China) and HRP bound Goat anti rabbit IgG (Proteintech, Wuhan, China).

For LRP1 CL II, CL IV domains and Gn (154–469 aa), the purified LRP1 domain and Gn (154–469 aa) were mixed under the same conditions, and then the SEC fractions were analyzed by SDS-PAGE.

### 2.3. Negative-Staining Sample Preparation

To prepare for negative staining, a 3% (*w*/*v*) uranyl acetate solution was centrifuged at 12000× *g* for 5 min to remove any undissolved particles. The solution was stored vertically at room temperature to ensure that it was not affected by vibration and light.

For sample preparation, the protein was diluted in the range of 0.005–0.1 mg/mL to determine the optimal concentration. A parafilm sheet was placed on the workbench and the carbon-coated grid underwent glow discharge for 15 s. Using tweezers, the grids were picked up and placed carbon-side up. After applying 3–5 μL of protein solution to the carbon side of the grid, it was incubated for 45 s. After incubation, the grid was gently blotted on filter paper to remove excess protein while ensuring that the edges were dry. Next, the grid was stained with uranyl acetate solution for 15 s, and the excess stains were blotted off.

The stained gridwas air dried for at least 2 min, then stored in the grid holder. Incomplete drying may lead to staining or corrosion, reducing the usable area of imaging.

### 2.4. Bio-Layer Interferometry Binding Assay

The binding kinetics between RVFV Gn (154–469 aa) and LRP1 CL domains were measured using Bio-Layer Interferometry (BLI) on an Octet RED96 system (ForteBio, Fremont, CA, USA). Purified Gn (154–469 aa) was biotinylated using biotinylation kit (Genemore, 1828M, Suzhou, China) and loaded to octet SA biosensor (Sartorius, Göttingen, Germany). The sensors were equilibrated in binding buffer (25 mM HEPES, pH 7.4, 150 mM NaCl, 0.02% DDM, 2 mM CaCl_2_/4 mM EGTA/6 mM MgCl_2_) prior to association measurements.

The following analytes were tested at serial dilutions: full-length LRP1, CL II (786–1165 aa), CL IV (3332–3779 aa), three truncated CL IV constructs covering repeats A21–A24 (3332–3491 aa), A25–A28 (3492–3649 aa), and A29–A31 (3652–3778 aa), and six point mutants in the truncated CL IV constructs (E3356A/D3357A/D3358A, D3374A/D3376A/D3378A, E3517A/D3518A/D3519A, D3556A/D3558A/D3560A, D3595A/D3597A/D3599A, D3633A/D3635A/D3637A).

SA Sensors labeled Gn protein were into analysis buffer to measure association for 300 s, followed by dissociation in same buffer for 300 s. Reference sensors without immobilized ligand were used for background value. Data were analyzed using ForteBio Data Analysis software (version 10; ForteBio, Fremont, CA, USA), and apparent equilibrium dissociation constants (KD) were calculated using a global 1:1 binding model.

For affinity analysis characterizing the interactions of the Gn head with full-length LRP1, truncated clusters, single repeat fusion protein and mutant forms. All experiments were performed in independent triplicates (*n* = 3) and values are reported as the mean ± standard deviation (SD) from the representative result.

For mechanistic validation assays, the protein to be detected was incubate with corresponding condition successively. Incubated protein was performed BLI detected the response change after each condition incubation. Using EGTA inhibition, Mg^2+^ ion replace and Ca^2+^ ion rescue, were performed to qualitatively confirm the reversibility and specificity of the calcium-dependent interaction.

### 2.5. AlphaFold3-Based Structural Modeling and Interface Analysis

The AlphaFold server was used to generate the structural prediction of the complex of LRP1 CL IV domain (3332–3779 aa) and Gn head domain (154–469 aa). The amino acid sequence was derived from UniProt entries [Q07954] and [P21401]. The sequence is submitted as a normal FASTA input without a user-defined structure template.

In order to accurately simulate the cation dependent structure, the entity type part (Entity type: ions; Name: Calcium) clearly includes four calcium ions, corresponding to the stoichiometric requirement binding motif of Ca^2+^. All forecasts are made using the default alphafold3 pipeline.

The server generates five structural models for each input, and selects the top model determined by the ranking credibility score for downstream analysis. The quality of the model was evaluated by the predictive local distance difference test (pLDDT) score and the predictive alignment error (PAE) matrix. In the selected model, the structured core region showed high confidence (pLDDT > 70–90).

UCSF ChimeraX (version 1.6.1; Resource for Biocomputing, Visualization, and Informatics, University of California, San Francisco, CA, USA), PyMOL (Schrödinger, LLC, New York, NY, USA), and the PDBePISA servers were used to visualize and analyze the predicted structure. The calculation and visualization of surface electrostatic potential use related tools to embed chimeras to characterize the interface charge complementarity. For interaction analysis, hydrogen bonds and salt bridges are determined using the distance cut-off value of 3.5 Å. All coordinate files generated by this study are provided in the Supplementary Materials.

## 3. Results

### 3.1. LRP1 Directly Interacts with the RVFV Gn Head in a Calcium-Dependent Manner

**Figure 1:**
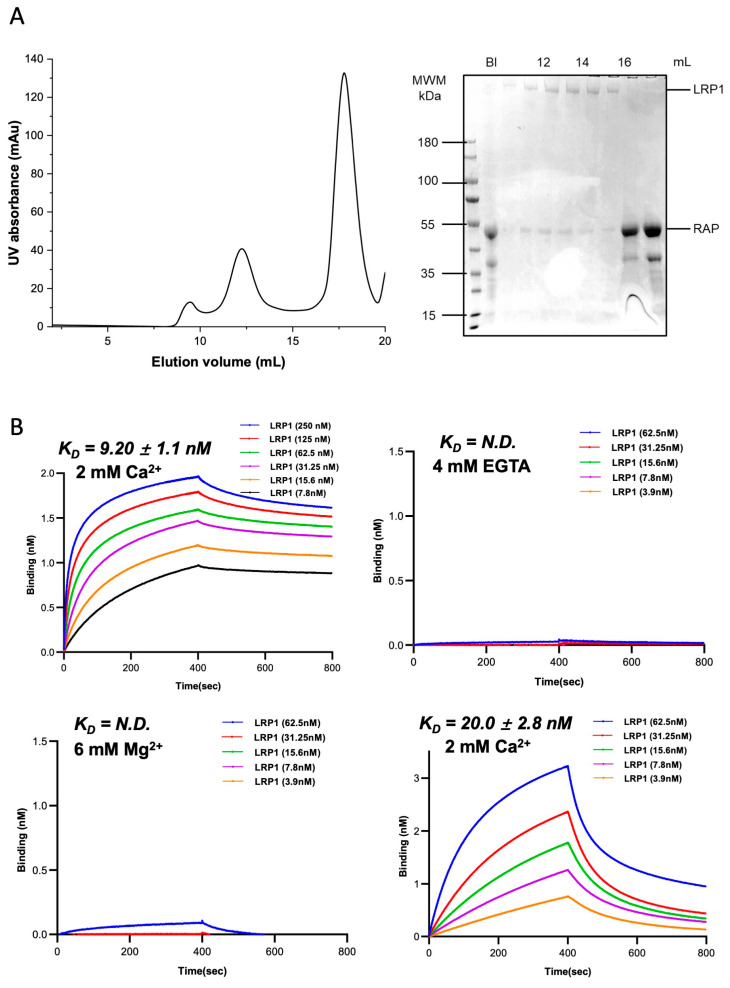
Biochemical analysis and binding kinetics of LRP1 and RVFV Gn. (**A**) SDS–PAGE analysis of purified full-length LRP1 protein. The gel shows the protein distribution of full-length LRP1 and RAP. The bands correspond to the expected molecular weights for each protein. (**B**) Binding kinetics of LRP1 and RVFV Gn. The color-coded curves show varying concentrations of RVFV Gn binding response with LRP1. Each curve represents the response during the association and dissociation phases. Data were fit to a 1:1 binding model to determine the apparent equilibrium dissociation constant (*K_D_*). Experiments were performed in independent triplicates (*n* = 3) for full-length LRP1 and its subdomains with the Gn head. BLI results showing the sequential effect of cation depletion and substitution. The binding signal is completely lost after the addition of 4 mM EGTA. Adding 6 mM MgCl_2_ to the EGTA-treated sample fails to rescue the interaction, suggesting that Mg^2+^ cannot replace Ca^2+^. In contrast, re-introducing 2 mM CaCl_2_ partially rescues the binding affinity.

Endogenous full-length LRP1 was purified from porcine lung tissue using the receptor-associated protein (RAP) as a folding chaperone. RAP ensures the correct assembly of LRP1 (Figure 1A). After two steps of affinity purification, functionally active LRP1 was obtained after removing RAP. This was confirmed by SDS-PAGE, which showed a single major band with the expected molecular weight (Figure S1). The purified receptor was used to check for direct binding to the RVFV envelope glycoprotein Gn using BLI.

To assess whether LRP1 directly recognizes the RVFV Gn, the purified receptor was also analyzed for binding to the recombinant Gn head domain using BLI. With 2 mM Ca^2+^ present, LRP1 showed a strong binding response by distinct association and dissociation phases (Figure 1B). It shows that the binding affinity (KD = 9.20 ± 1.1 nM) has a moderate to high interaction pattern, typical of ligand recognition observed within the LDL receptor family. Pre-incubation of LRP1 with EGTA completely blocked the BLI signal, suggesting that the removal of bound Ca^2+^ impacts the structural integrity required for ligand recognition. The subsequent addition of 6 mM MgCl_2_ did not rescue binding, indicating that Mg^2+^ cannot replace Ca^2+^ to support the interaction. Subsequently adding 2 mM CaCl_2_ to the Mg^2+^/EGTA-containing buffer, the binding response was partially rescued but still weaker than the initial Ca^2+^ condition. This partial recovery is consistent with residual Ca^2+^ buffering by EGTA and/or incomplete refolding of the Ca^2+^-coordinated modules, suggesting the structural necessity and specific selectivity of Ca^2+^ for maintaining LRP1’s ligand-binding pattern.

### 3.2. Both the CL II and CL IV Domains of Human LRP1 Interact with RVFV Gn in a Ca^2+^-Dependent Manner

**Figure 2:**
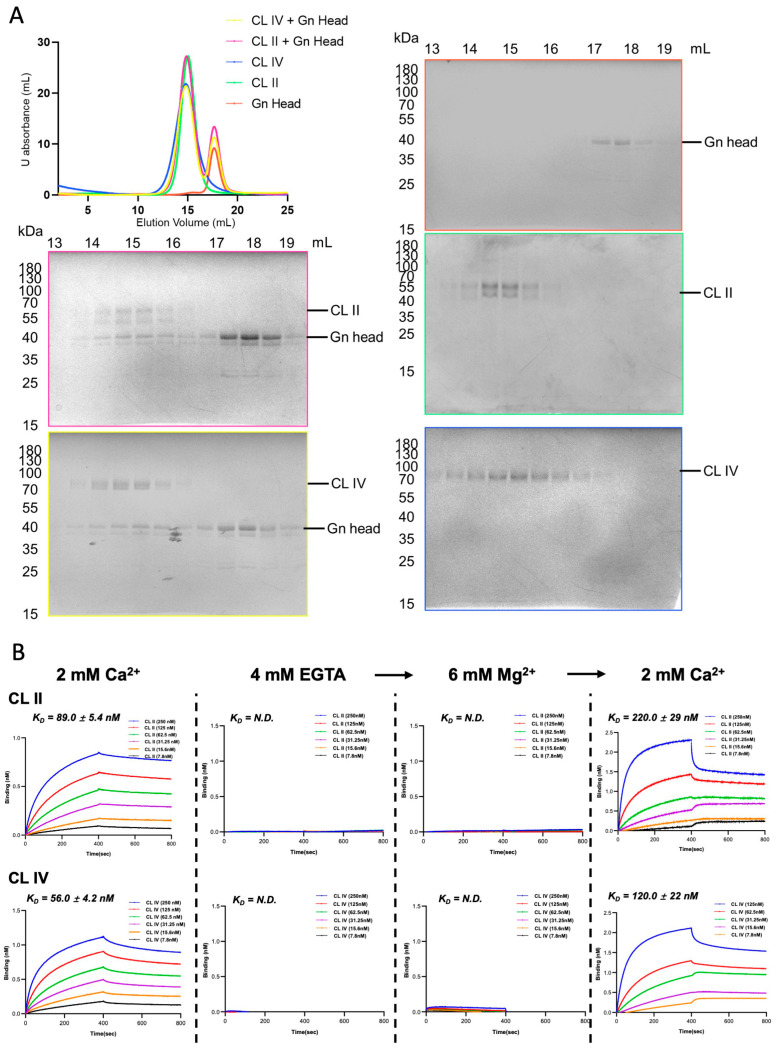
The CL II and CL IV domains of LRP1 interact with RVFV Gn in a Ca^2+^-dependent manner. (**A**) Size-exclusion chromatography (SEC) of purified CL II, CL IV, RVFV Gn Head, and its complex in the presence of 2 mM Ca^2+^. Both domains eluted earlier than expected when using a prestained protein ladder. When mixed with recombinant Gn, the SDS-PAGE of the corresponding fractions showed Gn co-elution, confirming complex formation. (**B**) BLI binding assays of CL II and CL IV with Gn under different cation conditions. In the presence of Ca^2+^, both domains showed concentration-dependent binding (*K_D_* = 89.0 ± 5.4 nM for CL II; 56.0 ± 4.2 nM for CL IV). Binding was blocked by EGTA and was not rescued by Mg^2+^, while the re-introduction of Ca^2+^ partially rescued binding response. Original images can be found in Supplementary Materials.

Based on previous reports identifying clusters II and IV (CL II and CL IV) as the principal ligand-binding regions of LRP1, these two domains were recombinantly expressed and purified for biochemical characterization. When analyzed individually via SEC in the presence of 2 mM Ca^2+^, both CL II and CL IV eluted earlier than expected when using a normal prestained protein ladder. This unexpected early elution time may be due to their elongated protein structure with tandem Ca^2+^-binding repeats and/or indicates a propensity for self-interaction. Upon mixing with the recombinant Gn head domain under the same Ca^2+^ conditions, the first chromatographic peak position showed no major shift; however, SDS-PAGE of the corresponding fractions showed co-elution of the Gn head with both CL II and CL IV(Figure 2A), thereby confirming complex formation in the solution.

To verify if the Ca^2+^ dependence existed in LRP1 individual clusters, BLI assays were performed under the same conditions (Figure 2B). In the presence of Ca^2+^, both CL II and CL IV produced clear, concentration-dependent association and dissociation phases, yielding binding affinities (*K_D_*) of 89.0 ± 5.4 nM (CL II) and 56.0 ± 4.2 nM (CL IV). 4 mM EGTA of Ca^2+^ chelation block binding affinity, suggesting that Ca^2+^ is indispensable. The subsequent addition of 6 mM MgCl_2_ did not rescue the major signal response, indicating that Mg^2+^ cannot substitute for Ca^2+^. When 2 mM CaCl_2_ was added to the Mg^2+^/EGTA-containing buffer, binding partially rescued but remained weaker (*K_D_* = 220 ± 29 nM for CL II; 120 ± 22 nM for CL IV) than in the initial Ca^2+^ condition. The results consistent with the Ca^2+^ dependence observed in the full-length LRP1.

Together, both CL II and CL IV specifically recognize RVFV Gn through a Ca^2+^ stabilized interface. Although each domain binds independently, their affinities are lower than that of full-length LRP1, suggesting that multivalent engagement of multiple clusters in the intact receptor contributes to the higher apparent affinity observed for the full-length Gn-LRP1 interaction.

### 3.3. Predicted Structures of Gn-CL IV Complexes Reveal Ca^2+^-Stabilized Multivalent Electrostatic Interfaces

Negative-stain electron microscopy (nsEM) (Figure S2) was employed to determine the overall structure of full-length LRP1, its clusters (CL II and CL IV), and their complexes with Gn or RAP. The nsEM micrographs showed monodisperse particles for all samples without apparent aggregation. Two-dimensional class averages of full-length LRP1 and LRP1-RAP displayed elongated structure, consistent with tandem complement type repeats, although individual domains were not well clear. Similarly, the CL II-Gn and CL IV-Gn complexes showed additional peripheral densities relative to their unbound forms, consistent with the presence of bound Gn. However, the particle boundaries and 2D averages appeared relatively blurred, likely due to particle flexibility and low contrast at this resolution. Despite the limited structural detail, the overall structure agreed with the predicted dimensions of the modeled complexes, confirming that the purified proteins were properly folded.

**Figure 3:**
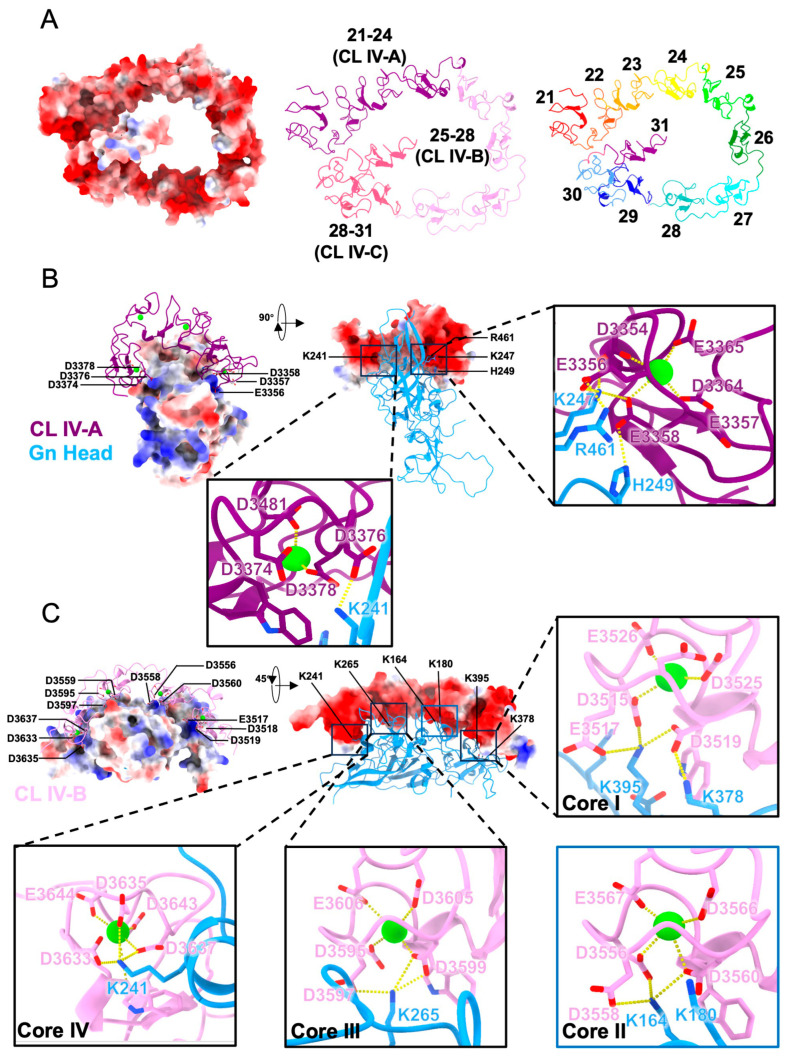
A predicted structure of the Gn-CL IV-B complex. (**A**) Overall predicted structure of CL IV and its electrostatic surface representation. Different segments and CR motifs are marked with different colors. (**B**) RVFV Gn (blue) in complex with CL IV-A (purple), highlighting the four Ca^2+^ cores (green spheres); electrostatic surface representation of CL IV-A and Gn, showing charge complementarity. Yellow dashed lines represent hydrogen bonds and salt bridges. (**C**) RVFV Gn (blue) in complex with CL IV-B (pink), highlighting the four Ca^2+^ cores (green spheres); electrostatic surface representation of CL IV-B and Gn, showing charge complementarity. Close-up views of the four main binding sites (Core I-IV), showing the Ca^2+^-coordinated acidic pockets and their interactions with Gn residues. Yellow dashed lines represent hydrogen bonds and salt bridges.

To investigate how LRP1 recognizes the RVFV Gn glycoprotein at the molecular level, structural models of the Gn head domain in complex with CL IV were generated using AlphaFold 3 (detailed in Section 2.5) (Figure S3) [24]. Each CR motif of CL IV adopted the β-hairpin fold that is stabilized by a single Ca^2+^ ion and coordinated by conserved acidic residues (Asp/Glu) pattern located in the C-terminal loop via sequence alignment (Figure S1B). For properly modeling available, CL IV was divided into three partially segments (CL IV-A:21–24, CL IV-B:25–28, and CL IV-C:29–31) that contained different CR motifs. The electrostatic surface representation showed that CL IV forms a continuous negatively charged groove that is complementary to the positive charge region distributed across the surface of the Gn head (Figure 3A,B). All predicted complexes displayed overall confidence scores within the pLDDT range of 70–90, supporting the structural property of the models. This predicted structure served as the molecular basis for our subsequent site-directed mutagenesis and BLI validation experiments. BLI experiments confirmed that CL IV-A and CL IV-B bind Gn in a Ca^2+^-dependent manner, whereas no binding affinity was detected for CL IV-C; therefore, no further analysis was conducted for this region.

The predicted structural model of the Gn-CL IV-A complex revealed that the 21–24 segment of LRP1 cluster IV adopts a curved conformation that is stabilized by four intramolecular Ca^2+^-binding cores. Two major cores are located at the terminal CR motifs and directly participate in Gn interaction (Figure 3B). Another two Ca^2+^ cores may serve a structural role which maintaining the rigidity and orientation of the acidic loops. Electrostatic surface mapping demonstrated charge complementarity between the negatively charged pockets of CL IV-A and the positively charged region on the Gn head domain surface. Based on a distance threshold of <3.5 Å, structural interaction analysis using PyMOL identified specific salt bridges and hydrogen bond interactions. These interactions are mainly driven by electrostatic attraction, hydrogen bond and salt bridge formation between oppositely charged residues, while Ca^2+^ ions play a structural maintain role. For clarity, only the most representative interactions are illustrated (Table S1). In the two terminal cores, acidic residues Asp3354–Glu3365 at one end and Asp3374–Asp3381 at another end. These two cores are constituting the major interface of interaction with RVFV Gn. At the N-terminal end, Glu3356 and Glu3358 form salt bridges with Gn residues Lys247, His249, and Arg461, whereas at the C-terminal end, Glu3376 establishes a salt bridge with Lys241. The central CR motif (22 and 23) remains solvent-exposed and make no direct contacts with Gn. Overall, the 21–24 fragment establishes leading dual-terminal cores and all cores participate in the electrostatic-driven interface. And the interaction supported by Ca^2+^-stabilized motif rigidity, which captured positive charged residues on the Gn surface through electrostatic interaction and multiple salt bridges. The PISA predicted a buried surface area (BSA) of ~998 Å^2^ [25,26], a characteristic of a specific yet moderate electrostatic interface dominated by salt bridges and charge complementarity.

The predicted structural model of the Gn-CL IV-B complex (repeat 25–28) shows four acidic pockets on the concave surface of CL IV-B that are each coordinated by one Ca^2+^ ion (Figure 3B). These cores, which are located under acidic loops, maintain stability by utilizing Ca^2+^ to coordinate several acidic residues (Asp/Glu). This Ca^2+^-mediated coordination is essential for driving the formation of stabilizing salt bridges with the Gn protein. Unlike CL IV-A models, all four Ca^2+^ ions in this complex directly stabilize the interface, enhancing both binding affinity and specificity. At Core I, the residues Asp3515–Glu3526 coordinate a Ca^2+^ ion, forming an acidic pocket. Asp3515, Glu3517, and Asp3519 form salt bridges with Gn Lys395 and Lys378. At Core II, the acidic residues Asp3555–Glu3567 also coordinate a Ca^2+^ ion, creating another acidic pocket. Asp3556, Glu3558, and Asp3560 form salt bridges with Gn Lys164 and Lys180. At Core III, Asp3605-Asp3599 coordinate a Ca^2+^ ion, forming the third acidic pocket, with Asp3597 and Asp3599 connect to Gn Lys265 through salt bridges. At Core IV, residues Asp3633–Asp3644 coordinate a Ca^2+^ ion, stabilizing the last acidic pocket, while Asp3633, Asp3635, and Asp3637 interact with Gn Lys241 via salt bridges.

The PISA analysis predicted a BSA of about 1613.4 Å^2^, which is significantly larger than typical electrostatic interfaces. This means that there is a strong and specific electrostatic interaction between CL IV-B and Gn. And the large BSA suggests that the interface is not only stable but also highly efficient in binding affinity.

### 3.4. Mutational and Domain Analyses Reveal That Ca^2+^ Coordination and Electrostatic Complementarity Cooperatively Mediate Gn–LRP1 Interaction

**Figure 4:**
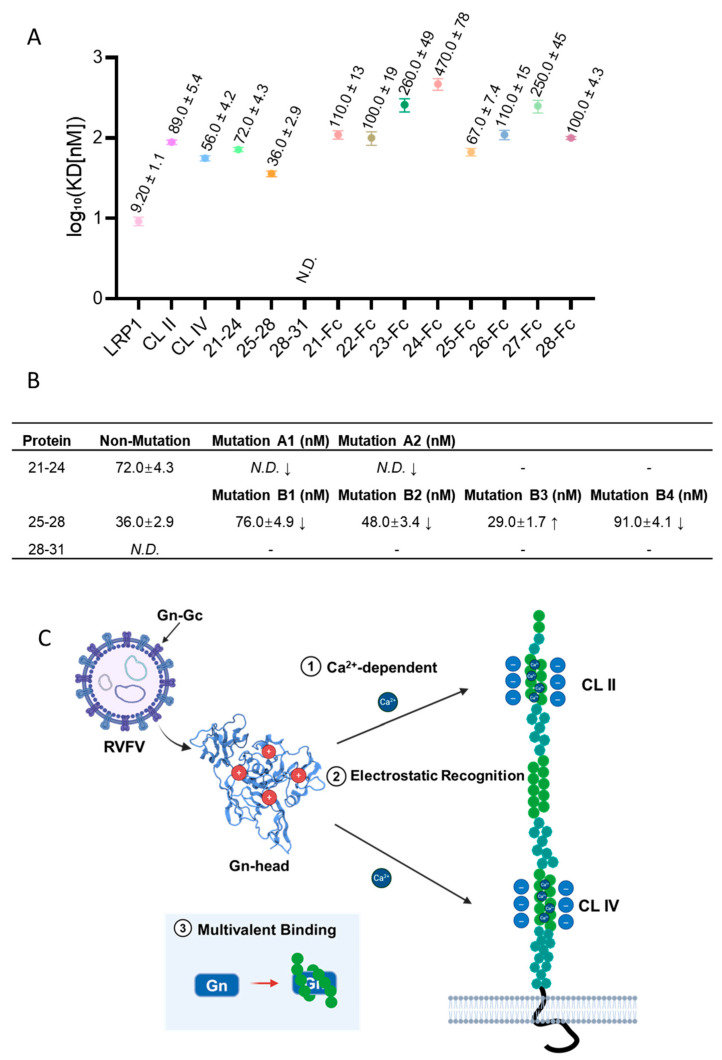
Structural and functional insights into LRP1–Gn interaction. (**A**) Comparison of the binding affinities of full-length LRP1, CLII, CLIV, and its segments with Gn. Full-length LRP1 shows the highest affinity. CLIV-B exhibits higher affinity than CLIV-A and CLIV-C. The CRs 25 and 28 may be contributing significantly to affinity. (**B**) BLI results of CLIV-A, -B, -C, and its mutated constructs. Mutations in the core regions of CLIV-A and CLIV-B cause a significant loss of affinity. (**C**) Putative working model for the interaction between RVFV Gn and the LRP1 receptor. The RVFV uses the calcium ion-dependent, electrostatic interaction-dominated receptor LRP1 for the entry host cell. This interaction can also enhance when the CRmotif comes into contact with glycoprotein, which leads to multivalent binding and increases the overall affinity.

The binding affinity of CL IV subsegments and their mutants were assessed under the same Ca^2+^ conditions using BLI (Figure 4A,B and Figure S4). The strongest binding affinity was detected with CL IV-B (CR25–28), which had a *K_D_* of 36.0 ± 2.9 nM, followed by CL IV-A (CR21–24) with a *K_D_* of 72.0 ± 4.3 nM. However, CL IV-C (CR28–31) showed no detectable affinity, suggesting that the major binding sites are located within CL IV-B and CL IV-A. Further analysis of individual CRs within these subdomains revealed that CR25 showed the highest binding affinity (Figure S5). And other CRs exhibited weaker binding affinity, supporting the hypothesis that the major binding sites are primarily located in the CR25–28 region of CL IV. This observation implies that the multivalent engagement of various clusters within the intact receptor enhances the apparent affinity associated with the Gn–LRP1 interaction.

This proposed model requires experimental validation. Possible strategies employing techniques such as cryo-EM or X-ray crystallography to visualize the structure of the receptor–virus complex and confirm the proposed multivalent binding mode.

To evaluate the role of acidic residues in these interactions, negative charge removal mutations were introduced at selected Ca^2+^-coordinated sites, based on hypothesis that altering the electrostatic pattern at these sites would affect binding affinity. In CL IV-A, mutations in the N-terminal acidic region (Glu3356Ala/Asp3357Ala/Asp3358Ala, Mutation A1) and the C-terminal acidic region (Asp3374Ala/Asp3376Ala/Asp3378Ala, Mutation A2). This mutated protein completely lost the binding affinity, with no detectable response in BLI (Figure 4B). This finding suggests that both acidic terminal regions are critical for Gn recognition, likely via their involvement in maintaining the electrostatic environment necessary for high affinity.

In CL IV-B, four mutations were analyzed to assess the role of specific acidic residues in protein-protein interaction mode (Figure 4B). Mutation B1 (Glu3517Ala/Asp3518Ala/Asp3519Ala) resulted in a *K_D_* of 76.0 ± 4.9 nM, which represents a more than twofold reduction in affinity compared to the non-mutated CL IV-B (*K_D_* = 36.0 ± 2.9 nM). In contrast, Mutation B2 (Asp3556Ala/Asp3558Ala/Asp3560Ala) exhibited a *K_D_* of 48.0 ± 3.4 nM, and Mutation B3 (Asp3595Ala/Asp3597Ala/Asp3599Ala) showed a *K_D_* of 29.0 ± 1.7 nM, both of which did exhibit moderate changes compared to the non-mutated type. This means that these mutations have a weak effect on binding affinity. However, Mutation B4 (Asp3633Ala/Asp3635Ala/Asp3637Ala) showed a two-threefold reduction in affinity (*K_D_* = 91.0 ± 4.1 nM). This indicates that specific residues within CL IV-B are significant for mediating the electrostatic interaction. And mutations B1 and B4 have the most obvious effects. In contrast, no detectable affinity was observed for CL IV-C with LRP1, suggesting that CL IV-C may just play a supporting role in the interaction.

All of these results indicated that multiple Ca^2+^-coordinated acidic residues are critical for the electrostatic interaction between Gn and LRP1. CL IV-A and CL IV-B have the major affinity sites, while CL IV–C contributes weakly to the interaction. The differential effects of mutations in these regions suggesting the importance of specific residues keep the multivalent electrostatic interface stable, which is essential for the efficient recognition of Gn by LRP1.

### 3.5. Proposed Model of the Gn-LRP1 Interaction

The biochemical and predicted structural data support a molecular mechanism in which RVFV Gn binds to clusters II and IV of LRP1 through acidic pockets that are coordinated with a Ca^2+^ ion. This establishes the initial electrostatic capture’s structural basis (Figure 4C). The positively charged region on the Gn (Lys/Arg clusters) surface align with the acidic grooves of CL IV to form the main interface. The nearby CR motif, which is best observed in CR25–28 (CL IV-B), adds serial contacts to make a multivalent, zipper-like arrangement that increases the overall binding affinity. In the CL IV A and B segment, the terminal acidic residues at the edges of CR21, 24, 25, and 28 act as a clamp, locking the Gn surface into the receptor’s groove and stabilizing the complex under Ca^2+^ conditions. Although CR29–31 (CL IV-C) shows no binding affinity when isolated, it may contribute indirectly by maintaining the local electrostatic environment and supporting the structure of nearby repeats, thereby enhancing the receptor–virus recognition.

## 4. Discussion

In this study, we examined the molecular mechanism by which the RVFV glycoprotein Gn interacts with its host receptor LRP1. Quantitative BLI analyses revealed that full-length LRP1 directly binds to the Gn head domain with nanomolar affinity in a Ca^2+^-dependent manner. Both CL II and CL IV of LRP1 could bind to Gn through Ca^2+^-stabilized acidic pockets. The CL IV exhibiting higher affinity than any single repeat, suggesting a multivalent and cooperative binding process. Predicted structural modeling revealed extensive charge complementarity between positive charged residues on Gn and acidic, Ca^2+^-coordinated pockets on LRP1. The mutagenesis of key acidic residues markedly reduced binding. These results define a Ca^2+^-stabilized, electrostatically driven, multivalent mechanism through which LRP1 recognizes RVFV Gn.

This binding mode is consistent with the conserved recognition paradigm of the LDL receptor (LDLR) family. The Ca^2+^-coordinated complement-type repeats (LA/CR modules) generate negatively charged grooves that capture positive charged residues on viral glycoprotein [23,27,28,29]. VLDLR recognizes the Semliki Forest virus through multiple LA modules [28]. LDLRAD3 engages the Venezuelan equine encephalitis virus using defined CRs [27], and VLDLR binds the Eastern equine encephalitis virus through several distinct binding modes [29]. These precedents suggest that Ca^2+^-dependent electrostatic interfaces between viral glycoproteins and LDLR-family receptors represent a general mechanism of virus–host interaction. Our findings extend this principle to a bunyavirus–LRP1 system and provide quantitative biochemical evidence and predicted molecular-level mappings of the interaction. It reveals the RVFV-receptor recognition and explains how multivalent engagement enhances apparent affinity.

The broad expression of LRP1 in hepatocytes and endothelial cells corresponds well with RVFV’s tropism and clinical pathology, suggesting that receptor distribution plays a role in tissue-specific infection [17,30,31]. The Ca^2+^ ion requirement observed here implies that variations in extracellular calcium levels or cellular ion homeostasis may modulate viral attachment efficiency. The interaction is driven by electrostatics, even small change in charge distribution, causing by viral mutations or host variation, could significantly influence infectivity. These mechanistic insights offer a basis for the design of antiviral agents that disrupt Ca^2+^ coordination or mask the acidic binding pockets of LRP1 to block receptor–mediated viral entry.

LRP1 is an endocytic receptor that plays a role in number of various physiological functions, including lipid metabolism, apoptotic cell clearance, and extracellular protease activity regulation. It also changes multiple signaling pathways by internalizing or scaffolding various ligands and co-receptors. This maintaining cellular homeostasis and regulating innate immune responses [18,19,20,32,33]. LRP1 signaling may interact with p38 mitogen-activated protein kinase (MAPK) cascades, which play a crucial role in stress and antiviral responses [32,33]. Moreover, the RVFV nonstructural protein NSs significantly inhibits host transcription and interferon induction, which are mechanistically reminiscent of the epigenetic silencing phenomena described in cancer cells [34,35]. Recent insights into cGAS-STING signaling in tumor immunity provide conceptual parallels: viruses and tumors both modulate this DNA-sensing axis to evade immune detection [36]. Thus, understanding LRP1-mediated viral entry may also know how host stress-response networks and chromatin regulation contribute to viral immune evasion.

The structural models provide insights into the interaction between RVFV Gn and LRP1. However, they are based on computational predictions from AlphaFold 3, which have inherent limitations. These models may not fully capture key details such as protein flexibility, especially in loop regions, or conformational changes that may occur during binding. Furthermore, AlphaFold 3 predictions do not consider the potential effects of glycosylation. This modification of glycoproteins like RVFV Gn head may influence the interaction structure and binding properties.

Because of these limitations, experimental validation through high-resolution techniques such as cryo-EM or X-ray crystallography is essential to validate the predicted models. These methods will provide a more accurate and detailed understanding of the structure, including the potential impact of glycosylation on the receptor–virus interaction. Additionally, this study did not consider the roles of other LDL receptor family members (such as LDLR and VLDLR) in RVFV entry. Future research ought to investigate their possible involvement and whether they act combined with LRP1.

Although we observed strong binding in vitro, the physiological relevance of this interaction in vivo remains uncertain. Further studies are needed to assess how factors like ion fluctuations and receptor availability affect viral entry in whole organisms.

## 5. Conclusions

This study provides molecular insights into the interaction between the RVFV glycoprotein Gn and its host receptor LRP1. We propose that the interaction is driven by a Ca^2+^-dependent “electrostatic clamp” mechanism that depends on Ca^2+^ and combines biochemical characterization with structural modeling based on AlphaFold 3. Our structural predictions identified negatively charged acidic pockets in LRP1 (CL IV) complementary to positively charged region on the Gn head domain. Site-directed mutagenesis and binding assays validated this model, confirming that calcium ions stabilize the acidic loops to facilitate essential salt bridges. This multivalent electrostatic complementarity explains the high affinity and specificity of the LRP1-Gn engagement. These findings expand our understanding of LDLR-family receptor recognition mechanisms and offer a structural framework for developing antiviral strategies targeting the Gn-LRP1 interaction.

## Data Availability

Any data or additional information required for re–analysis is also available upon request. Please direct requests to the corresponding author: Renhong Yan (yanrh@sustech.edu.cn).

## Supplementary Materials

The following supporting information can be downloaded at: https://www.mdpi.com/article/10.3390/biom16010014/s1, Figure S1: Purification and sequence alignment of LRP1 complement-type repeat modules; Figure S2: Negative-staining electron microscopy of purified RVFV Gn-LRP1 complexes; Figure S3: AlphaFold 3 structure predictions and confidence metrics for RVFV Gn-LRP1 models; Figure S4: Bio-layer interferometry analysis of RVFV Gn binding to LRP1 CL IV subsegments and their mutants; Figure S5: Bio-layer interferometry analysis of RVFV Gn binding to LRP1 complement-type repeat clusters; Figure S6: Uncropped and unedited original images of SDS-PAGE and Western Blot; Figure S7: Uncropped and unedited original images of CL II&IV-Gn SEC fractions SDS-PAGE; Figure S8: Uncropped and unedited original images of Gn head, CL II, CL IV SEC fractions SDS-PAGE; Figure S9: Uncropped and unedited original images of LRP1–RAP SEC fractions SDS-PAGE; Figure S10: Schematic overview of the experimental workflow to elucidate the RVFV Gn–LRP1 interaction mechanism; Table S1: Hydrogen bonds and salt bridges at the Gn Head-CL IV(21-28) interface.

## Abbreviations

RVFV: Rift Valley fever virus; WHO: World Health Organization; LRP1: Low-density lipoprotein receptor-related protein 1; BLI: Bio-layer interferometry; CL II: Cluster II; CL IV: Cluster IV; CRs: Complement-type repeats; RdRp: RNA-dependent RNA polymerase; GPC: Glycoprotein precursor; NSm: Nonstructural protein NSm; N: Nucleocapsid protein; NSs: Nonstructural proteins; LDLR: Low-density lipoprotein receptor; HEK293F: Human embryonic kidney 293F cells; PEI: Polyethyleneimine; SEC: Size-exclusion chromatography; SDS–PAGE: Sodium dodecyl sulfate–polyacrylamide gel electrophoresis; nsEM: Negative-stain electron microscopy; BSA: Buried surface area; MAPK: Mitogen-activated protein kinase; cGAS-STING: Cyclic GMP–AMP synthase–stimulator of interferon genes pathway.
