# Supplementary material for: LRP1 Interacts with the Rift Valley Fever Virus Glycoprotein Gn via a Calcium-Dependent Multivalent Electrostatic Mechanism
Source: Biomolecules. 2025 Dec 21;16(1):14. doi: 10.3390/biom16010014 (PMC12839305; doi:10.3390/biom16010014)
Supplement: Supplementary file 1 [file biomolecules-16-00014-s001.zip › biomolecules-3994136-supplementary.pdf]

Supplementary information

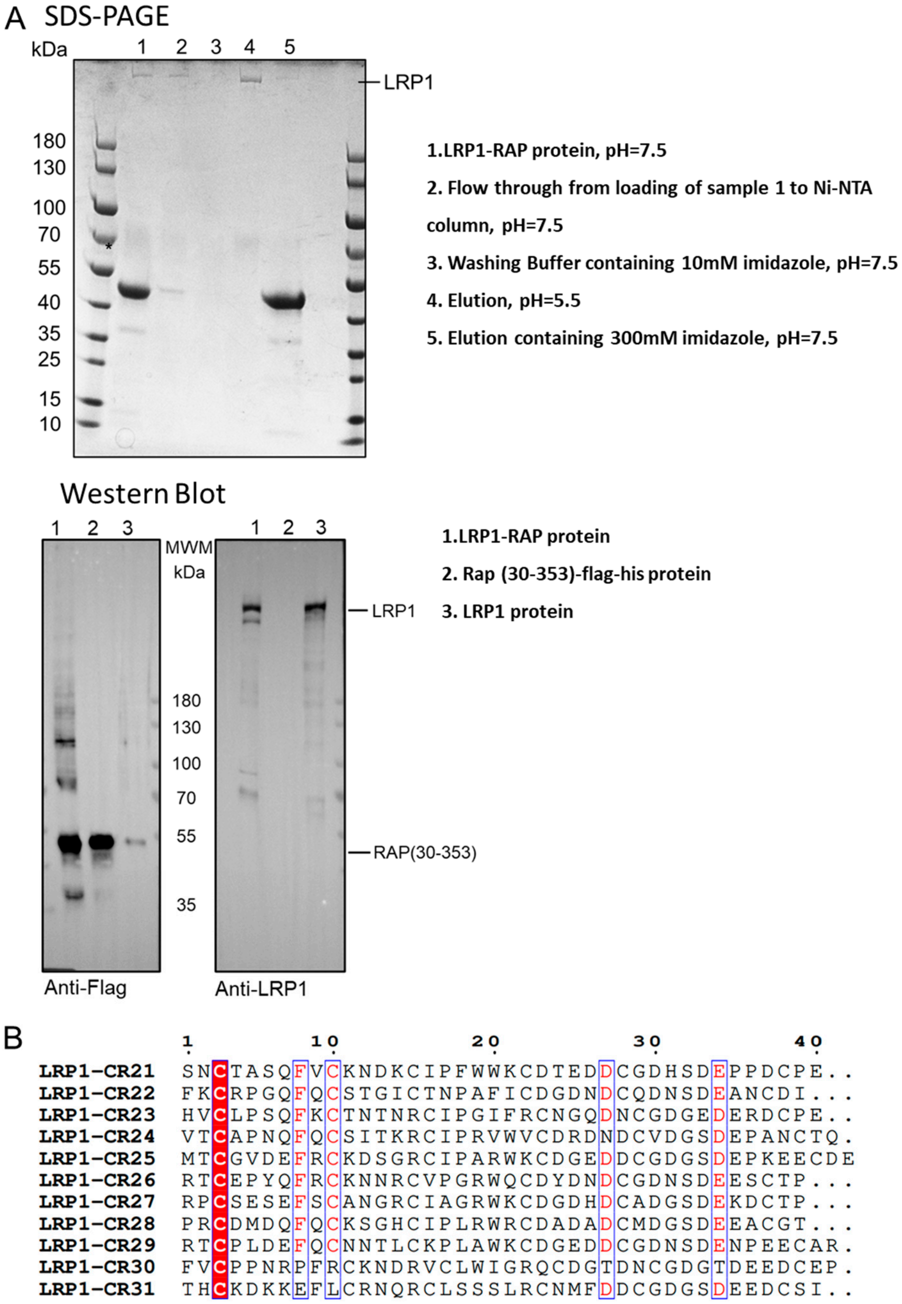

Figure S1. Purification and sequence alignment of LRP1 complement-type repeat modules

(A) SDS-PAGE and western blot analysis of purified LRP1 from porcine lung. Molecular weight markers (kDa) are indicated on the left.

(C) Multiple sequence alignment of LRP1 complement-type repeats CR21-CR31. Conserved cysteine residues (red) and calcium-binding acidic residues in C-terminal. These residues are essential for structural stabilization via disulfide bridges and Ca^{2+} coordination, both critical for ligand recognition and viral attachment.

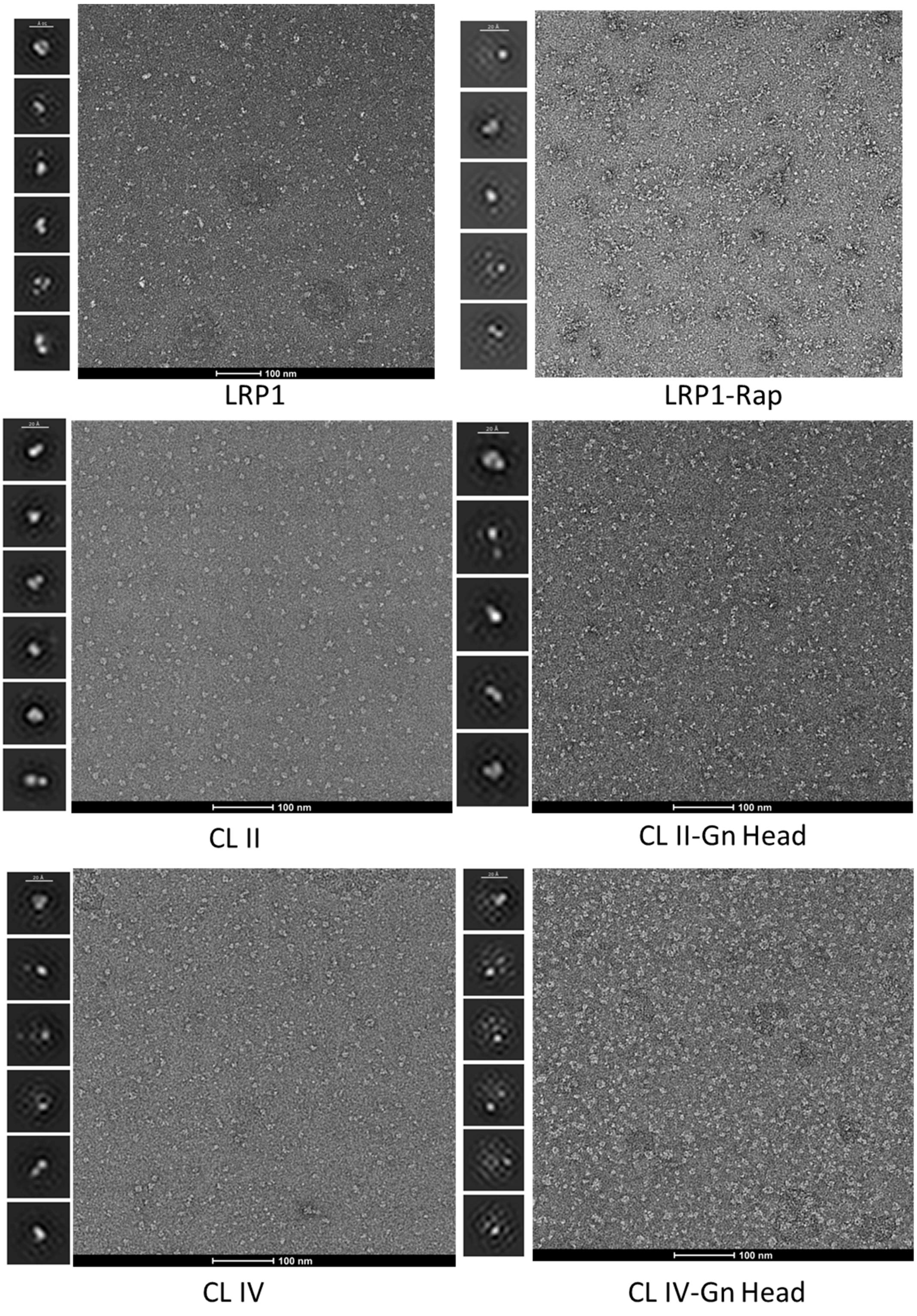

Figure S2. Negative-staining electron microscopy of purified RVFV Gn-LRP1 complexes

Representative negative-staining transmission electron microscopy (TEM) micrographs of purified Rift Valley fever virus (RVFV) Gn-LRP1 complex samples. Each panel shows distinct particle populations adsorbed onto carbon-coated copper grids and stained with 3% uranyl acetate. Insets display 2D class particle images highlighting the characteristic morphology.

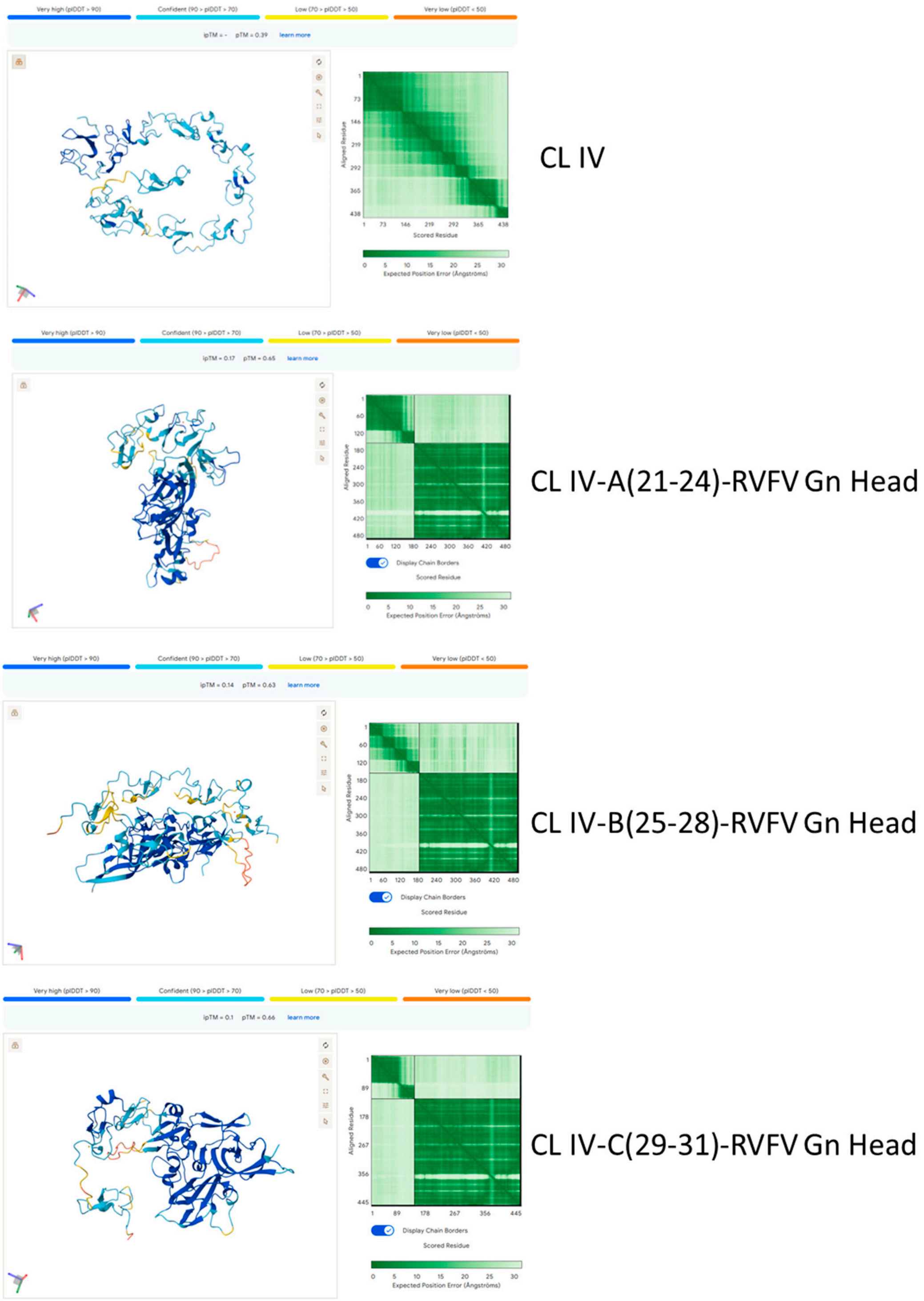

Figure S3. AlphaFold 3 structure predictions and confidence metrics for RVFV Gn-LRP1 models

Predicted structural models generated by AlphaFold 3 for individual and complexed domains of Rift Valley fever virus (RVFV) Gn and human LRP1 complement-type repeats (CR modules). Each panel shows the 3D predicted structure (left) colored by predicted local distance difference test (pLDDT) scores and the corresponding predicted aligned error (PAE) heat map (right), reflecting the relative spatial confidence between residues. Color scale indicates pLDDT confidence levels (blue, very high > 90; cyan, confident 70-90; yellow, low 50-70; orange, very low < 50).

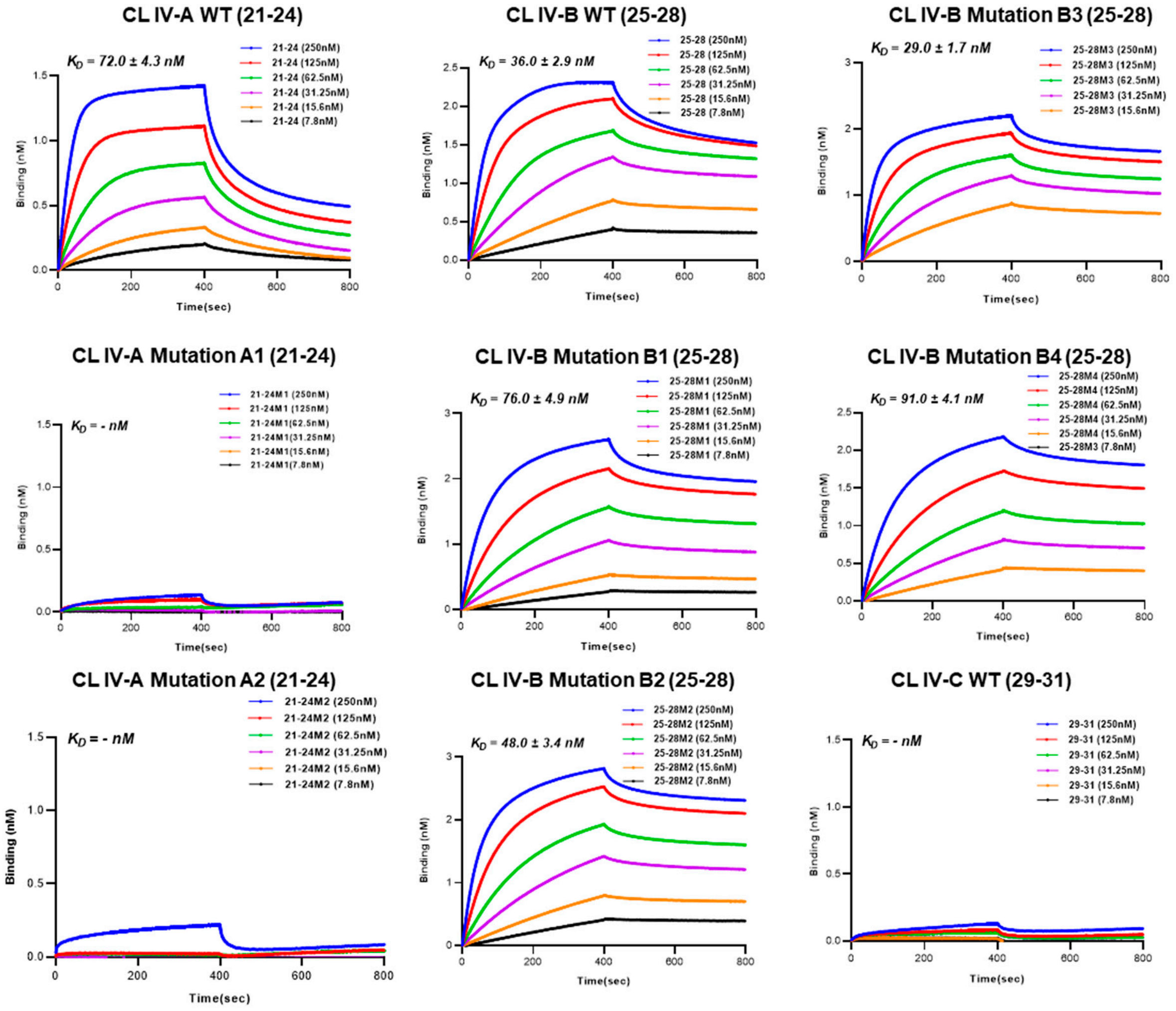

Figure S4. Bio-layer interferometry analysis of RVFV Gn binding to LRP1 CL IV subsegments and their mutants.

Representative BLI sensor grams showing real-time binding of recombinant RVFV Gn Head to various purified LRP1 CL IV subsegments and their mutants.

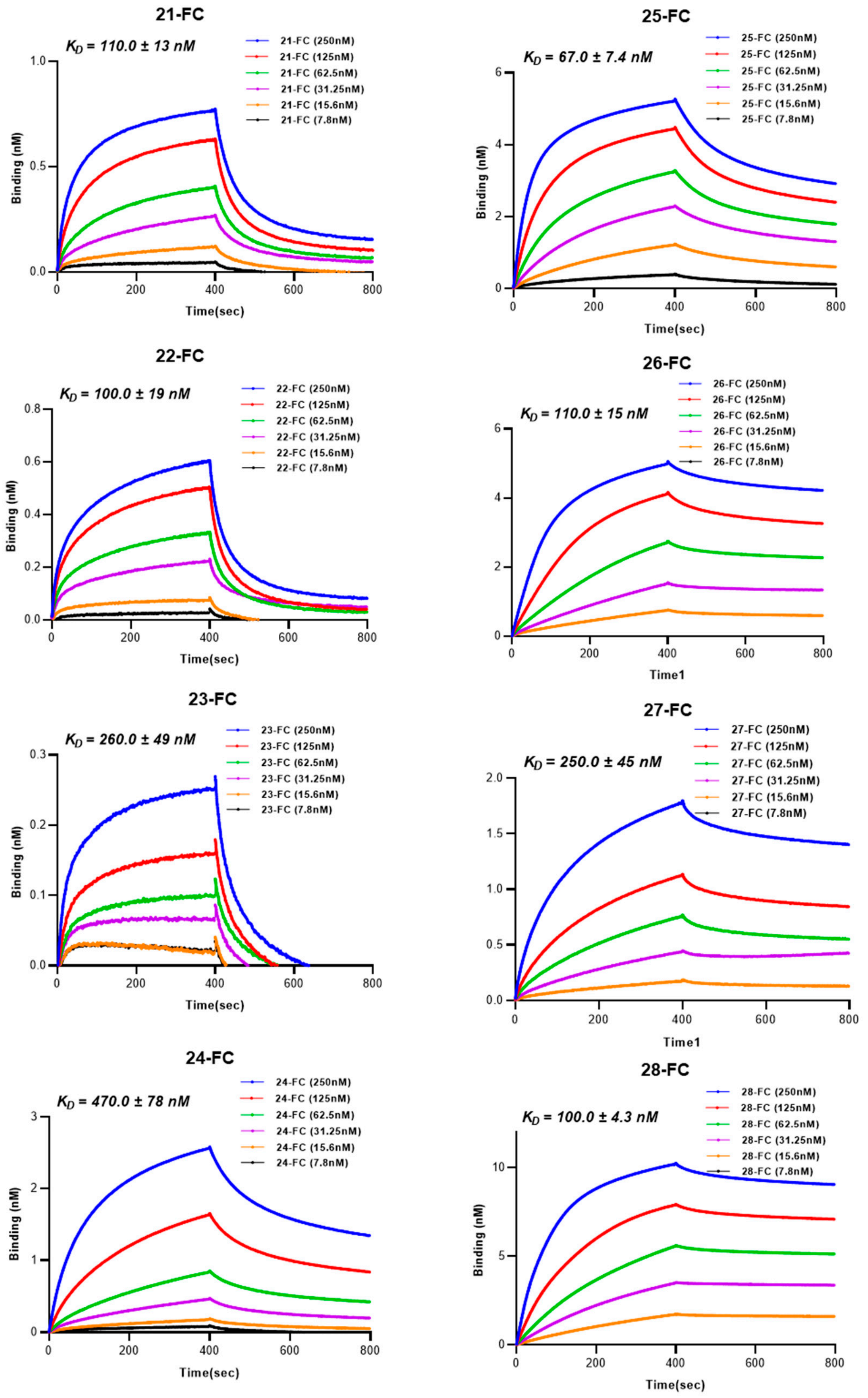

Figure S5. Bio-layer interferometry analysis of RVFV Gn binding to LRP1 complement-type repeat clusters

Representative BLI sensor grams showing real-time binding of recombinant RVFV Gn Head to various purified LRP1 complement-type repeat modules.

Table S1. Hydrogen bonds and salt bridges at the Gn Head-CL IV(21-28) interface

No.	Hydrogen Bond		Salt Bridge	
	CL IV-A&B (21-28)	RVFV Gn Head	CL IV-A&B (21-28)	RVFV Gn Head
1	ARG3465	HIS235	ASP3354	LYS247
2	ASN3333	THR288	GLU3356	ARG461
3	ASP3354	LYS247	GLU3356	HIS340
4	GLU3356	ARG461	GLU3356	LYS247
5	GLU3356	HIS340	ASP3358	LYS247
6	ASP3358	HIS249	ASP3358	HIS249
7	ASP3358	LYS247	ASP3474	LYS241
8	ASP3476	LYS241	ASP3476	LYS241
9	HIS3624	ASP225	HIS3598	ASP225
10	HIS3598	ASP225	ARG3511	GLU376
11	ARG3629	VAL326	ARG3506	GLU393
12	TRP3512	GLU376	ARG3552	ASP398
13	ARG3511	GLU376	ASP3515	LYS395
14	ARG3506	GLU393	GLU3517	LYS395
15	ARG3511	ASN431	ASP3519	LYS395
16	ASP3515	LYS395	ASP3519	LYS378
17	ASP3519	LYS378	ASP3519	LYS395
18	ASP3519	LYS395	GLU3537	LYS411
19	GLU3537	LYS411	ASP3556	LYS164
20	ASP3556	LYS164	ASP3558	LYS164
21	ASP3558	LYS164	ASP3560	HIS166
22	ASN3559	LYS180	ASP3560	LYS164
23	ASP3560	LYS164	ASP3560	HIS166
24	ASP3597	LYS265	ASP3597	LYS265
25	ASP3599	LYS265	ASP3599	LYS265
26	ASP3633	LYS241	ASP3633	LYS241
27	ASP3635	LYS241	ASP3635	LYS241
28	ALA3636	ASN237	ASP3637	LYS241
29	ASP3637	LYS241		

Uncropped gel figure

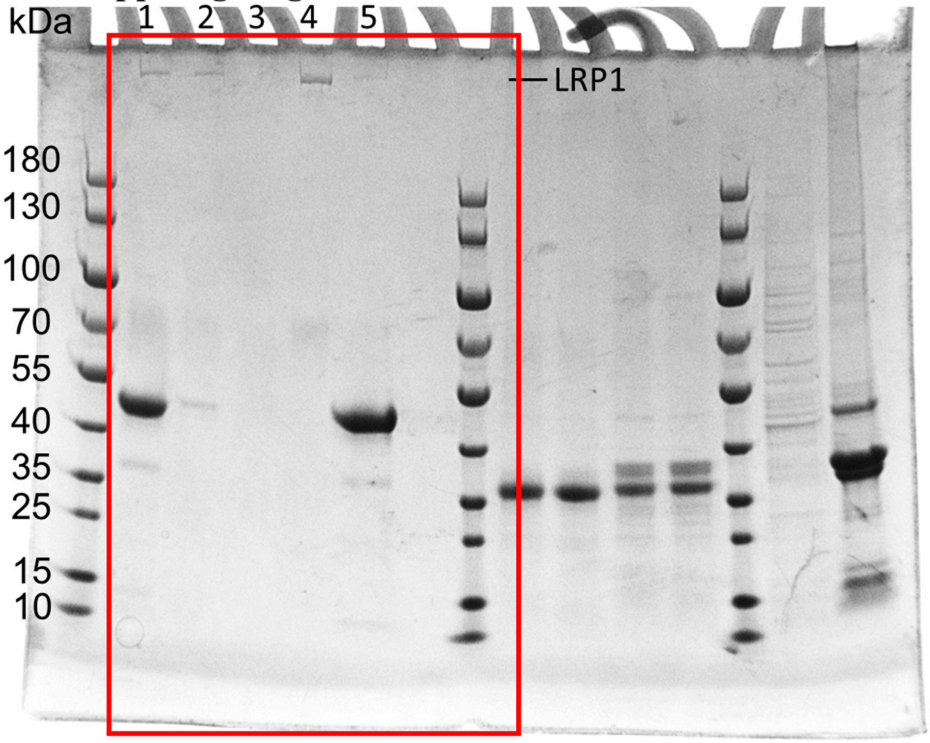

Uncropped Western blot figure

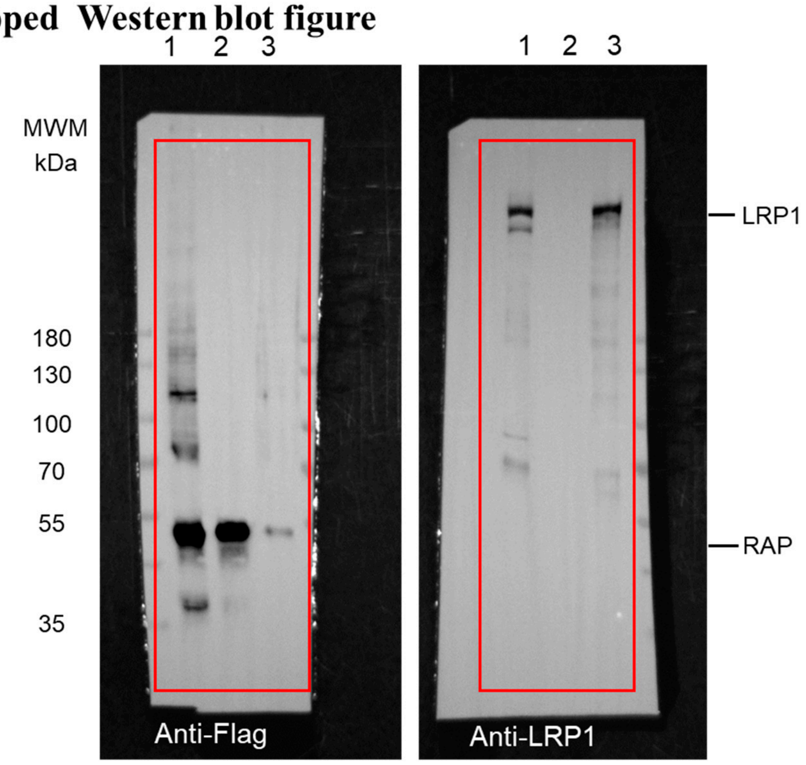

Figure S6 Uncropped and unedited original images of SDS-PAGE and Western Blot

The red box indicates the cropped area (Figure S1 A) after display.

Uncropped gel figure

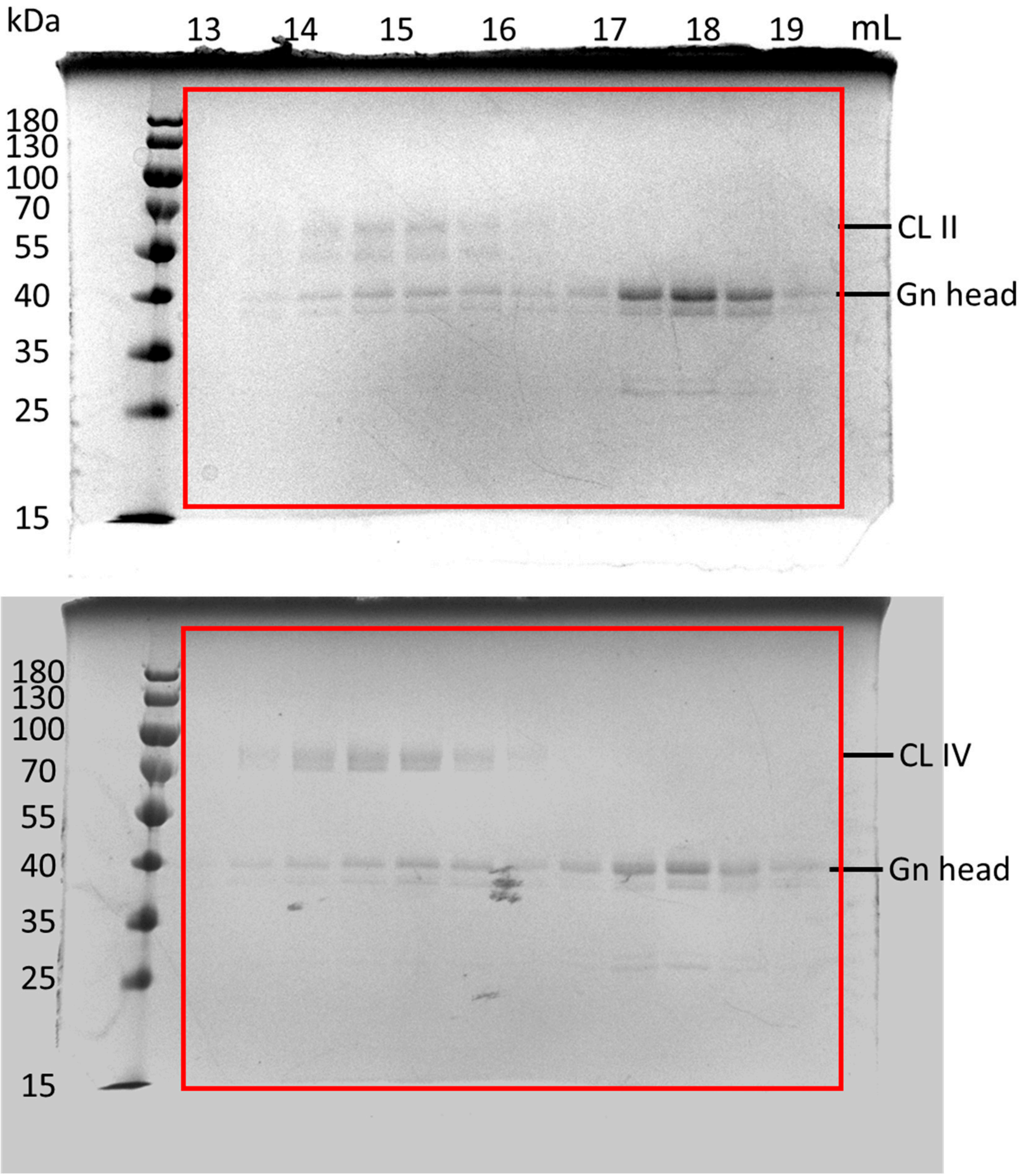

Figure S7 Uncropped and unedited original images of CL II&IV-Gn SEC fractions SDS-PAGE

The red box indicates the cropped area (Figure 2A) after display.

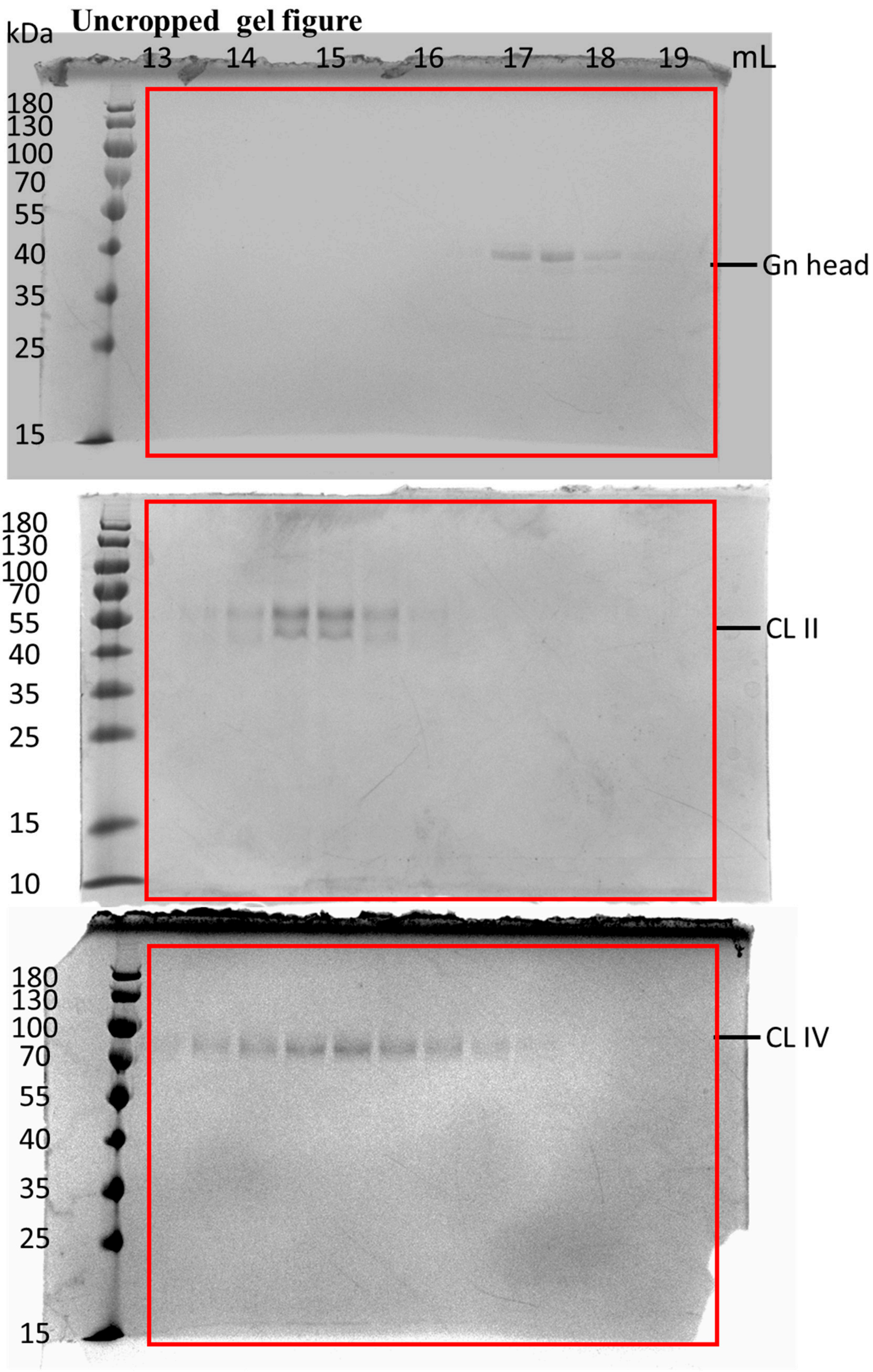

Figure S8 Uncropped and unedited original images of Gn head, CL II, CL IV SEC fractions SDS-PAGE

The red box indicates the cropped area (Figure 2A) after display.

Uncropped gel figure

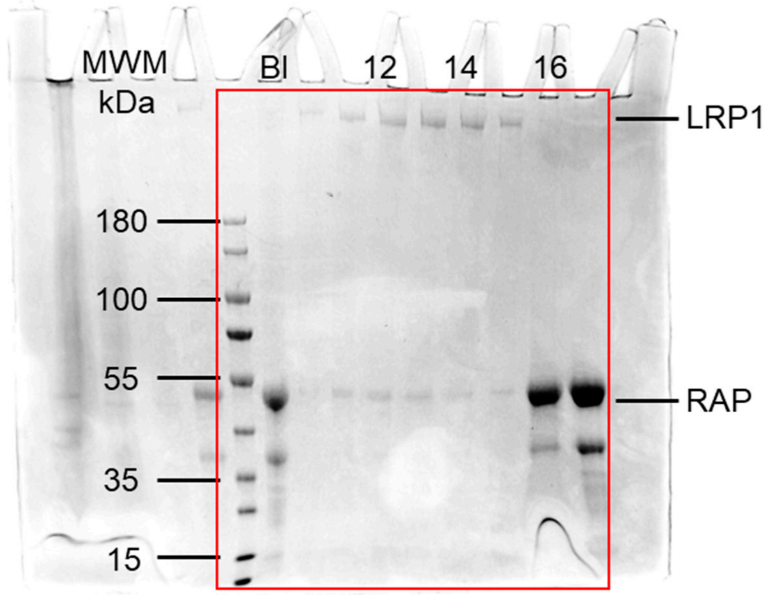

Figure S9 Uncropped and unedited original images of LRP1-RAP SEC fractions SDS-PAGE

The red box indicates the cropped area (Figure 1A) after display.

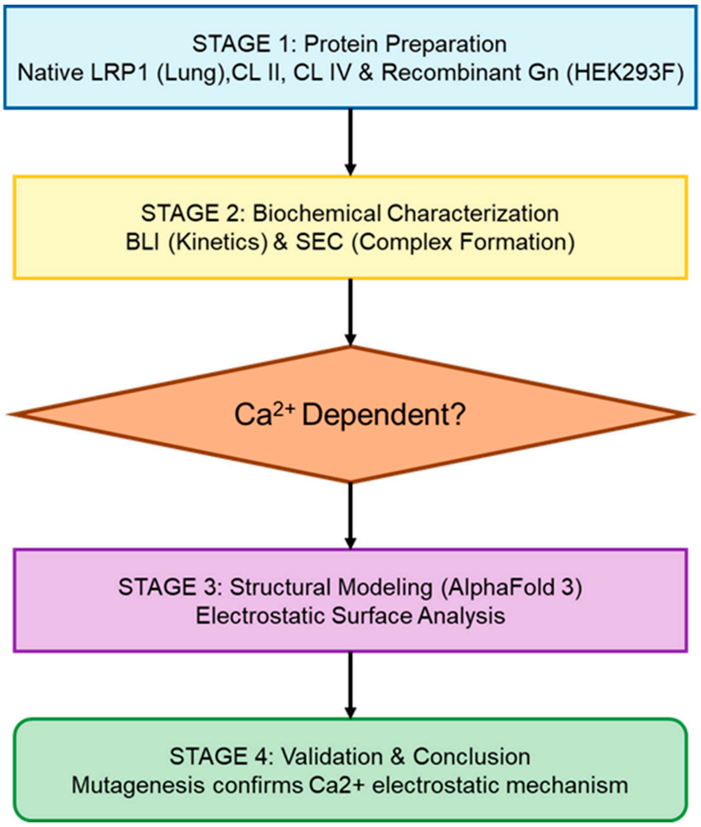

Figure S10 Schematic overview of the experimental workflow to elucidate the RVFV Gn–LRP1 interaction mechanism.

The study follows a stepwise approach integrating biochemical analysis with structural modeling:(Stage 1) Protein Preparation: Native full-length LRP1 was purified from porcine lung tissue, while the RVFV Gn head domain and LRP1 cluster domains (CL II, CL IV) were recombinantly expressed in HEK293F cells. (Stage 2) Biochemical Characterization: The direct interaction and complex formation were established using Bio-Layer Interferometry (BLI) and Size-Exclusion Chromatography (SEC), with a specific focus on identifying Calcium dependence. (Stage 3) Structural Modeling: Based on the identified Ca^{2+} requirement, AlphaFold 3 was employed to predict the binding interface and analyze electrostatic surface potentials. (Stage 4) Validation & Conclusion: The predicted "electrostatic clamp" mechanism was rigorously validated through site-directed mutagenesis of key Ca^{2+}-coordinating acidic residues, confirming the molecular basis of viral entry.
